# The Plant-Specific Dof Transcription Factors Family: New Players Involved in Vascular System Development and Functioning in *Arabidopsis*

**DOI:** 10.3389/fpls.2013.00164

**Published:** 2013-05-29

**Authors:** Rozenn Le Hir, Catherine Bellini

**Affiliations:** ^1^UMR1318 Institut Jean-Pierre Bourgin, INRA-AgroParisTech, INRA Centre de Versailles, Versailles, France; ^2^Department of Plant Physiology, Umeå Plant Science Centre, Umeå University, Umeå, Sweden

**Keywords:** transcription factors, DNA-binding with one finger, vascular system, *Arabidopsis*

## Abstract

In higher plants phloem and xylem are responsible for long-distance transport of water, nutrients, and signals that act systemically at short or long-distance to coordinate developmental processes. The formation of the plant vascular system is a complex process that integrates signaling events and gene regulation at transcriptional and posttranscriptional levels. Thanks to transcriptomic and proteomic analysis we start to better understand the mechanisms underlying the formation and the functioning of the vascular system. The role of the DNA-binding with one finger (Dof TFs), a group of plant-specific transcription factors, recently emerged as part of the transcriptional regulatory networks acting on the formation and functioning of the vascular tissues. More than half of the members of this TF family are expressed in the vascular system. In addition some of them have been proposed to be mobile proteins, suggesting a possible role in the control of short- or long-distance signaling as well. This review summarizes the current knowledge on Dof TFs family in *Arabidopsis* with a special focus on their role in vascular development and functioning.

## Introduction

The gradual colonization of the land through plant evolution has been possible thanks to the differentiation of complex vascular tissues that provide mechanical support and allow long-distance transport of water and nutrients. In land plants, vascular tissues are comprised of two conducting tissues the phloem and the xylem, and intervening cambium that can generate the different conducting cell types (Scarpella and Helariutta, 2010). Whereas the xylem, whose vessels are dead cells with highly lignified walls, ensures the transport of water, mineral salts, and some hormones, the phloem controls the transport and distribution of photosynthetic products from leaves to meristems and other sink organs such as fruits, tubers, and roots. The sieve elements (SE) are the phloem conducting cells and are associated with one or a few CCs. In general the meristematic mother cell of a CC-SE complex divides unequally in the longitudinal axis. One daughter cell develops into one or several metabolically hyperactive CCs with a dense cytoplasm and numerous mitochondria. The second one goes through a controlled disintegration process and differentiates into the SE, which is a highly specialized and unique developmental program (Juergensen et al., 2003). In the last 15 years, major insights have been obtained in the identification of the regulatory actors controlling the establishment of the vascular system as well as the fate of the vascular cell types (reviewed in Zhou et al., 2011). Many transcription factors including bHLH, HD-ZIP, ARF-GAP, AT-Hook, and Dof proteins families were shown to be involved, suggesting the existence of complex transcriptional regulatory networks in which transcription factors are likely to physically interact as shown recently by several bHLH and Dof TFs (Zhang et al., 1995; Kang and Singh, 2001; Skirycz et al., 2008). Some of these families of transcription factors are conserved among eukaryotes but the Dof TFs family, which is the focus of this review, is of particular interest for being specific to plants.

**Figure 1:**
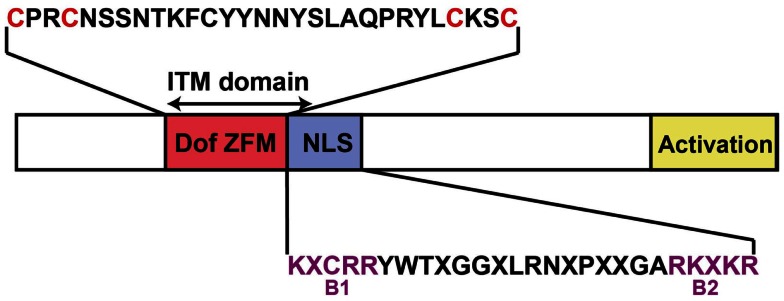
**Schematic representation of an *Arabidopsis* Dof transcription factor structure**. The Dof *Z*ing *F*inger *M*otif (Dof ZFM), the nuclear localization signal (NLS), and the activation domain are indicated in pink, purple, and yellow respectively. The cysteine residues for putative coordination of zinc are shown in red letters in the Dof domain amino acid sequences. The consensus sequences of the bipartite NLS (B1 and B2 basic regions in purple letters) are based on Krebs et al. (2010). X represents any amino acids. The double-ended arrow indicated the intercellular trafficking motif (ITM) that confers the capacity to mediate selective intercellular trafficking (Chen et al., 2013). This motif includes the ZFM and the first motif of the bipartite NLS.

The first *D*NA-binding with *o*ne *f* inger (Dof) transcription factor has been identified in maize (ZmDof1) and was shown to be involved in light response and transcriptional regulation of genes involved in carbon metabolism (Yanagisawa and Sheen, 1998; Yanagisawa, 2001). Dof TFs are plant-specific and are characterized by a particular zinc finger domain, comprising a conserved region of 50 amino acids with a C_2_–C_2_ finger structure, associated to a basic region, that binds specifically to DNA sequences with a 5′-(A/T)AAAG-3′ core (Figure 1). A particular feature of these TFs is that the nucleus localization signal (NLS), which directs Dof proteins to the nucleus in *Arabidopsis thaliana* (Krebs et al., 2010), is an atypical bipartite NLS with a 17 amino acid long linker between its flanking basic regions (Figure 1). This bipartite NLS is highly conserved in plant Dof transcription factors (Krebs et al., 2010) and its feature allowed identifying *Dof* genes in a various set of plant species (for details, see below). Along plant evolution, Dof transcription factors might have originated from a common ancestor, likely represented by the single *Chlamydomonas reinhardtii* gene, and then expanded in the different taxonomic groups of vascular plants through recurrent duplication events (Moreno-Risueno et al., 2007). In Gymnosperms and lower plants (*Selaginella moellendorffii* and *Physcomitrella patens*) so far analyzed eight to nine *Dof* TF genes are found (Moreno-Risueno et al., 2007). In Angiosperms an average of 30 genes are found with 27 in *Brachypodium distachyon* (Hernando-Amado et al., 2012), 30 in rice (*Oryza sativa*) (Gaur et al., 2011), 36 *Dof* genes in *Arabidopsis* (reviewed in Yanagisawa and Schmidt, 1999; Moreno-Risueno et al., 2007), 37 in tomato (*Solanum lycopersicon*) (Cai et al., 2013), or 41 in poplar (*Populus trichocarpa*) (Yang and Tuskan, 2006), indicating additional recent duplications in higher plants (Moreno-Risueno et al., 2007). This diversification appears to be associated to the establishment and differentiation of the vascular system during the same period of time, which raises the question whether members of this plant-specific transcription factor family are involved in the formation of the vascular system or its functioning. Indeed besides their involvement in the regulation of different processes such as carbon assimilation (Yanagisawa, 2001; Tanaka et al., 2009), light signaling (Park et al., 2003; Ward et al., 2005; Gabriele et al., 2010), seed development or germination (Papi et al., 2000; Gualberti et al., 2002; Dong et al., 2007; Rueda-Romero et al., 2012), flowering (Sawa et al., 2007; Fornara et al., 2009), stomata functioning (Gardner et al., 2009; Negi et al., 2013), or response to phytohormones (Kang and Singh, 2001; Kang et al., 2003; Nakano et al., 2006) most of them are strikingly expressed in the vascular tissues suggesting a function in long-distance signaling (Gualberti et al., 2002; Papi et al., 2002; Ward et al., 2005; Fornara et al., 2009; Rueda-Romero et al., 2012). Recently several Dof TFs were shown to have a central role in the development of the vascular system (Konishi and Yanagisawa, 2007; Guo et al., 2009; Gardiner et al., 2010; Kim et al., 2010). Based on these observations it is possible to speculate that the plant-specific Dof TFs are likely to regulate directly or indirectly a wealth of processes associated to the development of the vascular system or to its role in long-distance signaling. In the present review we aim at summarizing the current knowledge about the Dof TF family highlighting their role in the control of vascular development and functioning.

## All *Arabidopsis* Dof TFs are Potentially Expressed in the Vascular Tissues

In *Arabidopsis thaliana*, out of the 36 Dof TFs, 20 have been found expressed in the vascular tissues (Table 1 and references there in). *In silico* studies also indicate the presence of *cis*-acting elements potentially driving specific expression in the vascular tissues in the promoter of these genes. In plants, mainly through 5′-deletion experiments, several positive *cis*-acting promoter elements have been identified to drive vascular-specific expression of reporter genes. The study of the rice tungro bacilliform virus (RTVB) present only in the phloem of infected plants allowed the identification of a virus promoter functional in plants (Bhattacharyya Pakrasi et al., 1993; Yin and Beachy, 1995). This promoter contains four domains allowing a strong phloem-specific expression (Yin et al., 1997a,b): a GATA motif which had already been shown to be implicated in light dependent phloem-specific gene expression in plants (Lam and Chua, 1989); an ASL box characterized by the presence of a specific GCA direct repeat sequence present only in phloem-specific promoters and missing in xylem-specific promoters; a Box II containing CCA/TGG repeat and a CCCC sequence that because of their presence in the promoter of plant genes expressed in vascular tissue might be important *cis*-elements for expression in vascular tissues. These three *cis*-elements, Box II, the ASL box, and the GATA motif were shown to act in combination (synergistically and/or additively) to confer phloem-specific expression (Yin et al., 1997a). Similarly the study of the coconut foliar decay virus (CFDV) led to the identification of a 13 bp motif sequence (Hehn and Rohde, 1998) which is highly conserved in several phloem-specific promoters as reported by Yoshida et al. (2002). Finally, the 26 bp RSE regulatory element was found to drive a vascular-specific expression of the *GRP1* (*Glycine-rich protein1*) (Keller and Baumgartner, 1991). All these motifs act synergistically or additively and Yin et al. (1997a) have suggested that at least two of these motifs are required for phloem expression. More recently, Ruiz-Medrano et al. (2011) identified degenerate sequences containing CT/GA- and GT/GA-rich repeats within many of the promoters of 150 *Arabidopsis* genes homologous to members of the pumpkin phloem transcriptome. Interestingly the CT/GA motifs resemble cognate sites for the Dof family of transcription factors, which bind to the core sequence AAAG/CTTT (Higo et al., 1999; Yanagisawa and Schmidt, 1999).

**Table 1:** ***Arabidopsis Dof* genes phylogeny and nomenclature**.

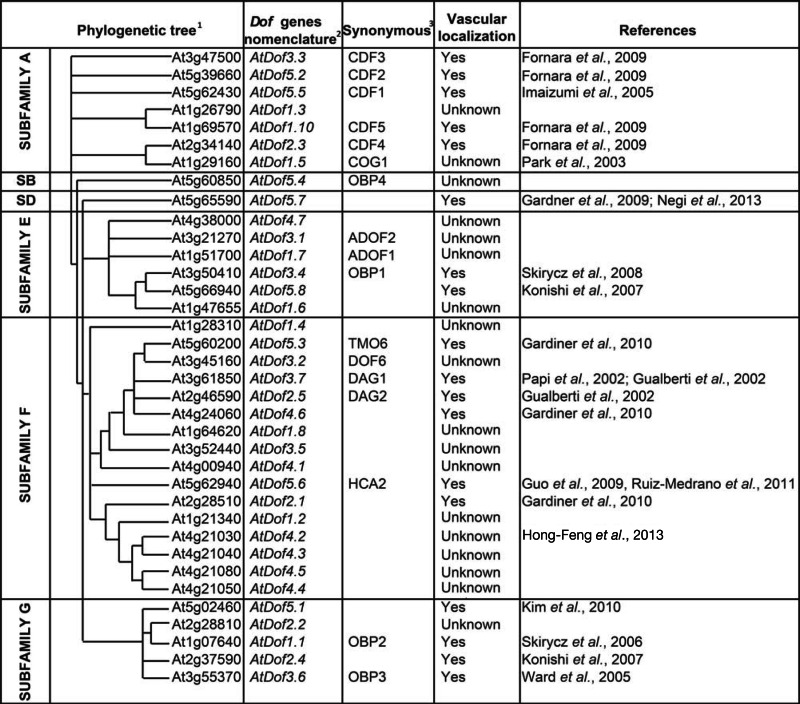

*^1^Phylogenetic tree of the Arabidopsis Dof gene family according to Moreno-Risueno et al. (2007). ^2^Dof genes nomenclature according to Yanagisawa (2002). ^3^The original names used in publications or for registration in a database*.

Searching for these *cis*-elements in the 1000 bp upstream sequences of the *Arabidopsis* Dof TF family members we showed that at least two of these vascular-specific motifs were found in the promoter region of 31 *Dof* genes display (Table 2). Among the remaining five, the promoter of *AtDof2.1* and *AtDof1.8* genes contain three and five GATA motifs respectively and the promoter of *AtDof4.4* contains two ASL boxes (Table 2). These observations suggest that the vast majority, if not all, of *Arabidopsis* Dof transcription factors are likely expressed in the vascular system (Table 2).

**Table 2:** **Synthesis of the predicted vascular-related regulatory *cis*-elements motifs in *AtDof* promoters**.

*AtDof* genes	Predicted Regulatory c/s-Elements^1^					Transcriptomic databases^2^		
	Box II motif	ASL box motif	GATA motif	13 bp motif	RSE motif	Ph	PC	VB
*AtDofl.1*	1	–	1	–	–			
*AtDof1.2*	–	2	4	–	–			
*AtDof1.3*	–	4	5	–	–			
*AtDof1.4*	–	1	6	1	–			
*AtDof1.5*	–	2	6	–	–			
*AtDof1.6*	–	–	1	–	–			
*AtDofl.7*	–	–	1	–	–			
*AtDof1.8*	–	–	5	–	–			
*AtDofl.10*	–	–	1	2	–			
*AtDof2.1*	–	–	3	–	–			
*AtDof2.2*	–	3	5	–	–			
*AtDof2.3*	–	2	4	–	–			
*AtDof2.4*	–	1	5	–	–			
*AtDof2.5*	–	2	3	–	–			
*AtDof3.1*	–	–	1	–	–			
*AtDof3.2*	–	1	2	–	–			
*AtDof3.3*	–	1	2	1	–			
*AtDof3.4*	–	1	2	–	–			
*AtDof3.5*	–	2	4	1	–			
*AtDof3.6*	–	2	3	–	–			
*AtDof3.7*	–	–	4	–	–			
*AtDof4.1*	–	–	4	1	–			
*AtDof4.2*	–	1	2	–	–			
*AtDof4.3*	–	1	1	–	–			
*AtDof4.4*	–	2	–	–	–			
*AtDof4.5*	–	2	2	–	–			
*AtDof4.6*	–	1	2	1	–			
*AtDof4.7*	–	1	5	–	–			
*AtDof5.1*	–	1	3	–	–			
*AtDof5.2*	–	–	3	1	–			
*AtDof5.3*	–	1	1	2	–			
*AtDof5.4*	–	1	3	–	–			
*AtDof5.5*	–	4	2	–	–			
*AtDof5.6*	–	1	2	1	–			
*AtDof5.7*	–	–	2	–	1			
*AtDof5.8*	–	2	4	–	–			

*^1^Box II motif: **TGGNCCCCN** (Yin et al., 1997a,b); ASL box: **GCA** N(20) **GCA** (Yin et al., 1997a); GATA motif: **W**NMN**GATA** (Gilmartin et al., 1990; Yin et al., 1997a); 13bp motif: **ATAAG**X**A**XXXX**GA** (Yoshida et al., 2002); RSE motif: **G**XXXXXX**ACTTTC**X**T**X**T** (Hatton et al., 1995; Hauffe et al., 1993; Keller and Baumgartner, 1991)*.

*^2^AtDof genes found in the transcriptomic databases related to the vascular system of Arabidopsis (Birnbaum et al., 2003; Zhao et al., 2005: Mustroph et al., 2009; Gandotra et al., 2013), Poplar (Schrader et al., 2004), and Celery (Vilaine et al., 2003)*.

*Grey color indicates the tissue in which the gene has been found to be expressed*.

*Ph, phloem; PC, procambium; VB, vascular bundle*.

The mining of RNA profiling datasets at the resolution of the vascular tissue (phloem, cambium, xylem) and vascular cell type (CC, SE) (Hertzberg et al., 2001; Birnbaum et al., 2003; Vilaine et al., 2003; Schrader et al., 2004; Zhao et al., 2005; Lee et al., 2006; Brady et al., 2007; Ruiz-Medrano et al., 2011; Gandotra et al., 2013) further indicate an expression of most *Dof* genes in the vascular system. Indeed, the expression of *Dof* TF genes could be found, either in the xylem, or the phloem or both (Table 2). *AtDof1.1*, *AtDof2.2*, *AtDof3.2*, *AtDof3.6*, *AtDof3.7*, *AtDof5.3*, and *AtDof5.5* were identified in the global phloem cells transcript databases established in *Arabidopsis* (Birnbaum et al., 2003; Zhao et al., 2005). In addition poplar orthologs of *AtDof1.6*, *AtDof3.4*, *AtDof4.6* were found in the transcriptomic analysis of the wood forming region (Hertzberg et al., 2001; Schrader et al., 2004) and the celery orthologs of *AtDof1.8* and *AtDof4.6* were expressed in the phloem of celery (Vilaine et al., 2003), suggesting that *AtDof1.6* and *AtDof1.8* which promoter contains one and five GATA motifs respectively could also be expressed in the vascular region. Besides these genes and the ones already experimentally described as expressed as well in the vascular system (Table 1), the recent transcriptome analysis of microdissected provascular/procambial cells or complete vascular bundle by Gandotra et al. (2013) allowed identifying new *AtDof* genes (*AtDof1.3*, *AtDof1.4*, *AtDof1.7*, *AtDof1.8*, *AtDof2.2*, *AtDof3.1*, *AtDof4.5*, and *AtDof4.7*) potentially expressed in vascular tissue at different step of its development.

In conclusion it is likely that almost all the members of the *Arabidopsis* Dof TFs family are at least expressed in the vascular system, being so far one of the TF family displaying such a strong tissue-specificity.

## *AtDof* Transcription Factors are Potential Mobile Transcription Factors

Coordinated growth and development of multi-cellular organisms require mechanisms that allow for extensive cell-to-cell communication which can be mediated by signaling molecules directly transported from one cell to the other. Sessile nature of plant growth also necessitated the evolution of mechanisms that rapidly transmit signaling molecules in response to environmental changes or pathogen attacks. These mobile signals in plants can be proteins, RNAs, small RNAs, and other small molecules. They are transmitted through the vascular system in a long-distance (between organs), or through plasmodesmata in a short distance (between cell types) (Lucas et al., 2009; Dinant and Suárez-López, 2012).

**Figure 2:**
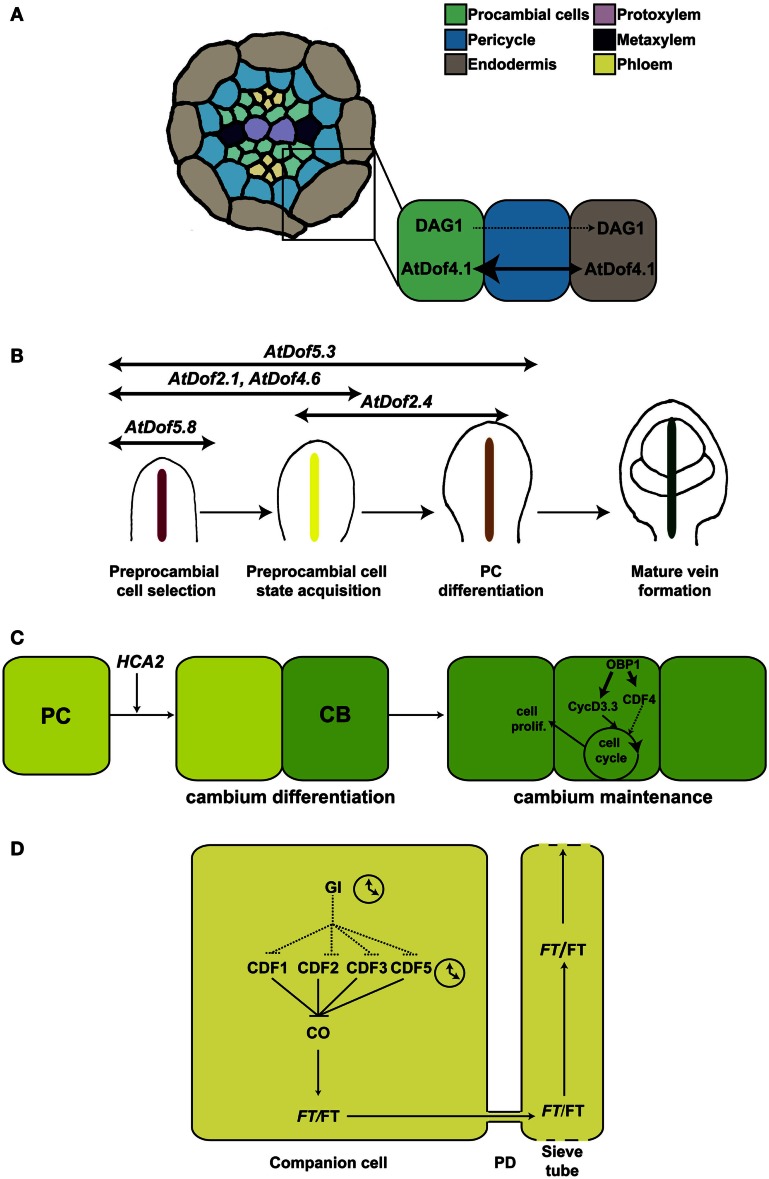
**The Dof TFs are involved in different steps of plant development**. **(A)** Non-cell-autonomous function of DAG1 and AtDof4.1 in the root. Schematic representation of an *Arabidopsis* root showing the different cell types. The black box focuses on stele, pericycle, and endodermis in which the cell-to-cell movement of DAG1 (Lee et al., 2006) (dashed line) and AtDof4.1 (Chen et al., 2013) (plain line) occur. The arrow and its size indicate the direction and the intensity of the protein movement respectively. **(B)** Pattern of expression of several Dof TFs at early stage of formation of the leaf vascular tissues. The PIN1 expression domain defines the preprocambial cell selection zones (purple). Then the preprocambial cell state acquisition is controlled by the expression of *ATBH-8* and *SHR* transcription factors (yellow). The preprocambial cells will give rise gradually to the procambium (PC) cells (brown) that will differentiate into phloem and xylem tissues and form the mature vein (green). The involvement of the *AtDof* s gene in these processes is essentially based on the colocalization of their expression patterns and those of the already known regulators PIN1 and ATHB-8. **(C)** Hypothetic role of Dof TFs for cell cycle during cambium formation/maintenance. The transcription factor AtDof5.6/HCA2 has been suggested to be involved in the transition between the procambium (PC) and the cambium (CB), and the AtDof3.6/OBP3 is supposed to control cambium maintenance through the control of the cell cycle. **(D)** Schematic representation for the role of CFD1, 2, 3, and 5 on the GI/CO/FT signaling pathways. In the phloem companion cell some of the steps of the photoperiodic flowering pathway are taking place. The core of this pathway is composed of GIGANTEA (GI), CONSTANS (CO), and FLOWERING LOCUS T (FT). In the sieve tube, the arrow indicates the movement of FT/*FT* as well as the sap flux direction. The clock symbol next to *GI* and *CDF* genes indicates that their expression is under the control of the circadian clock. PD, plasmodesmata.

By comparing the translational and transcriptional patterns of several TFs, including Dofs, expressed in a cell-type specific manner in *Arabidopsis* root, Lee et al. (2006) found that the translational pattern was broader or different than the transcriptional pattern for two *Dof* TFs, *AtDof3.7/DAG1* and *AtDof4.1*, suggesting that these TFs could move out from the cell/tissue they were expressed. In the case of *AtDof4.1* the transcriptional expression domain was contained in the translational one indicating that the expansion in the expression pattern must occur after the synthesis of the mRNA (from pericycle to endodermis, Figure 2A). Thus it is likely that it occurred via cell-to-cell protein movement, as shown for the CAPRICE protein (Kurata et al., 2005). For *AtDof3.7/DAG1*, because the transcriptional *AtDof3.7_prom_:GFP* pattern (in the stele, Figure 2A) was shown expressed in a different tissue compared the one described in the published root mRNA expression map (Birnbaum et al., 2003) the protein might not be expressed in its native tissue. Nevertheless, the expansion of the pattern is unlikely to be because of mRNA movement since the mRNA and the translational expression pattern were also different (transcriptional and translation fusions expressed in the stele and endodermis cell layer respectively; Figure 2A) (Lee et al., 2006).

In addition, cell-type specific expression maps from *Arabidopsis* root and cell-type specific translatome data were produced by several groups (Birnbaum et al., 2003; Nawy et al., 2005; Lee et al., 2006; Brady et al., 2007; Mustroph et al., 2009) and represent an extremely valuable source of information for predicting putative mobile transcription factors. By analyzing these data sets Lee and Zhou (2012) further proposed that seven *Dof* TFs (*AtDof1.1*, *AtDof2.1*, *AtDof2.2*, *AtDof2.4*, *AtDof3.2/DOF6*, *AtDof3.6/OBP3*, *AtDof3.7/DAG1*) are potential mobile transcription factor RNAs.

A direct experimental proof of the intercellular movement of a non-cell-autonomous Dof TFs has recently been brought by Chen et al. (2013) who showed that AtDof4.1/IDT1 (INTERCELLULAR TRAFFICKING DOF 1, IDT1) moves between the root stele and the endodermis in a plasmodesmata-selective pathway. This movement seems to be possible in either ways but more efficient from the endodermis to the stelar tissues (Figure 2A) (Chen et al., 2013). Moreover, the authors demonstrated that IDT1 movement requires an intercellular trafficking motif (ITM) comprising the Zinc Finger motif and the nuclear localization signal (NLS (Figure 1) (Chen et al., 2013). By looking for the existence of an ITM sequence in the different members of the Dof TF family, Chen et al. (2013) also showed that in contrast to AtDof5.4 and AtDof2.2, AtDof3.7/DAG1, and AtDof4.5 were also able to move from cell to cell.

In conclusion, some of the Dof TFs are able to move from cell to cell, however, although several *Dof* genes have been found expressed in the phloem, there is so far no evidence for long-distance transport since neither RNAs nor Dof proteins have been found in transcriptome and proteome datasets of phloem sap (Giavalisco et al., 2006; Deeken et al., 2008; Kehr and Buhtz, 2008).

## AtDof Transcription Factors are Involved in the Control of Vascular Development

### Control of the procambium formation during leaf development

Vascular development is initiated by the procambium formation, which is followed by the differentiation of the procambium into phloem and xylem. It is now well established that in the leaf primordia the procambium formation begins with the specification of undifferentiated ground meristem cells, by still unknown positioning information, that follows redirection of polar auxin flow to distinct convergent points by the auxin efflux carrier AtPIN1 (Scarpella et al., 2006). The *ATHB-8* gene [a Class III homeodomain-leucine zipper (HD-ZIP III)], a differentiation-promoting transcription factor of the vascular system (Baima et al., 1995), follows the expression of *AtPIN1* and the consequent restricted routes for auxin transport that define the sites of procambium formation (Scarpella et al., 2006), defining ATHB-8 as one of the earliest marker for the acquisition of procambial cell identity. Recently, several Dof transcription factors were shown to be expressed during the very early steps of procambium formation. Konishi and Yanagisawa (2007) showed that the GUS expression driven by the *AtDof5.8* promoter was similar to that of *PIN1* at very early stages of vascular development either in embryos, young leaf primordia, roots, or flower buds. These observations suggest that *AtDof5.8* might play a role in the *de novo* formation of procambium from ground meristem cells and in the very early processes of vasculature formation (Konishi and Yanagisawa, 2007) (Figure 2B). The same authors showed that the timing of expression of *AtDof2.4*, in contrast to *AtDof5.8*, was quite similar to that of the *ATHB-8* gene during procambium formation (Baima et al., 1995; Scarpella et al., 2004) suggesting that *AtDof2.4* might play a role in the processes following the primary formation of procambium in various organs (Figure 2B). *AtDof2.1*, *AtDof4.6*, and *AtDof5.3* are expressed in the root vascular system (Birnbaum et al., 2003) but recently, Gardiner et al. (2010) showed that during leaf development they are also expressed in overlapping subepidermal domains with a comparable dynamics. Expression of *AtDof2.1* and *AtDof4.6* was sustained at all stages of vein formation, while that of *AtDof5.3* stopped during procambium differentiation. The expression domains of *AtDof2.1*, *AtDof4.6*, and *AtDof5.3* overlapped with that of *ATHB-8* expression, suggesting that these three Dof TFs are expressed at preprocambial stages (Gardiner et al., 2010). *AtDof2.1*, *AtDof4.6*, and *AtDof5.3* expression was always initiated in wide domains that became laterally confined over time while *ATHB-8* expression domains are always narrower and comprised in the *Dof* expression domain. In addition the fact that discrete *AtDof2.1*, *AtDof4.6*, and *AtDof5.3* expression domains do not show *ATHB-8* expression may suggest that the expression of *AtDof2.1*, *AtDof4.6*, and *AtDof5.3* is initiated prior to the acquisition of the *ATHB-8* preprocambial cell state (Figure 2B) (Gardiner et al., 2010).

## *AtDof* Genes Control Vascular and Interfascicular Cambium Formation and Activity

The vascular cambium is the lateral meristem source of secondary xylem and secondary phloem. The fascicular cambium, initiated from the procambium, is present between the xylem and the phloem of a vascular bundle. Additionally an interfascicular cambium can also appear during secondary growth, giving rise to interfascicular fibers in the floral stem in species like *Arabidopsis*. By studying the *Arabidopsis* gain-of-function mutant *high cambial activity2* (*hca2*), which shows a precocious formation of the interfascicular cambium and its subsequent cell division, Guo et al. (2009) showed that the phenotype was due to the overexpression of *AtDof5.6* gene. Repression of AtDof5.6/HCA2 activity led to the disruption of interfascicular cambium formation and development in inflorescence stems. HCA2 promotes interfascicular cambium formation at a very early stage of inflorescence stem development. In addition Guo et al. (2009) showed that *AtDof5.6/HCA2* is preferentially expressed in the vasculature of all the organs, but more particularly in the cambium, the phloem, and the interfascicular parenchyma cells of inflorescence stems. In the *hca2* mutant high cambial activity and high phloem proliferation activity were detected not only in inflorescence stems, but also in petioles and leaf major veins suggesting that, in addition to interfascicular cambium formation/development, *AtDof5.6*/*HCA2* may also be involved in other developmental aspects during vascular tissue development, for instance, (pro)cambium initiation and activity (Figure 2C).

Additionally two *Dof TFs* (*AtDof3.4/OBP1* and *AtDof2.3*) are suspected to be involved in the control of the maintenance of the cambium and/or cambial activity by controlling the cell cycle (Skirycz et al., 2008) (Figure 2C). *AtDof3.4/OBP1* is highly expressed in tissue with high cell proliferation activity (cell cultures, calli, developing embryo, apical, and root meristems, fascicular procambium in the stem), and while looking for direct targets of *AtDof3.4/OBP1* several core cell cycle genes and transcription factors were identified (Skirycz et al., 2008). OBP1 modulates cell cycle activity by affecting the expression of CDKA regulators, S-phase specific transcription factors and components of the replication machinery, and is therefore important for cell cycle onset. OBP1 most likely operates through a direct regulation of *CYCD3.3* and *AtDof2.3* gene expression since it was shown to interact physically with their respective promoter of *AtDof2.3* and *CYCD3.3* (Skirycz et al., 2008) (Figure 2C).

## AtDof TFs are Involved in the Regulation of Long-Distance Signaling

### AtDof TFs regulate the photoperiodic flowering response

In order to adapt to the seasonal changes, plants synchronize their developmental program to be able to flower during the longest days of the year. The molecular genetics studies have allowed identifying actors involved in this photoperiodic flowering pathway. The circadian-clock controlling flowering signaling pathway takes place in the phloem CC, and recruits *GIGANTEA* (*GI*), *CONSTANS* (*CO*), and *FLOWERING LOCUS T* (*FT*) genes (Kobayashi and Weigel, 2007; Turck et al., 2008). The clock protein CO, which is expressed in the CC, induces *FT* transcription, and the FT protein moves long-distance through the phloem sap to reach the meristem where it triggers flowering (Corbesier et al., 2007). Additional actors in this pathway were identified by screening a library of *Arabidopsis* transcription factors systematically expressed in CC (Imlau et al., 1999; Fornara et al., 2009). These transcription factors, that all belong to the subfamily A of Dof TFs family (Moreno-Risueno et al., 2007), were named CYCLING DOF FACTOR (CDF), suggesting that this clade would almost be exclusively involved in flowering response (Table 1). Five *Dof* TFs genes (*AtDof5.5*/*CDF1*, *AtDof5.2*/*CDF2*, *AtDof3.3*/*CDF3*, *AtDof2.3*/*CDF4*, and *AtDof1.10*/*CDF5*) have been shown to be expressed in the vasculature throughout the plant and were shown to negatively regulate the transcription of *CO* (Imaizumi et al., 2005; Fornara et al., 2009) (Figure 2D). Although these *Dof* TFs were not shown to move in the phloem sap, they indirectly act on long-distance signaling by regulating the expression of *CO*.

### AtDof TFs contribute to the regulation of light signaling

Yanagisawa and Sheen (1998) reported for the first time the role of a Dof TF in light signaling. They showed that the mRNA level of the maize gene *ZmDof1* and its ability to bind DNA were regulated by light. In *Arabidopsis*, four *Dof* TFs have been suggested to be involved in photomorphogenesis processes like germination and hypocotyl growth (Papi et al., 2000, 2002; Gualberti et al., 2002; Park et al., 2003; Ward et al., 2005). During seed germination, light is required to convert the inactive Pr form of phytochrome into the active Pfr that will trigger the germination. In this context, the knockout mutant in *AtDof3.7/DAG1* requires substantially less phytochrome signaling than the wild type to germinate (Papi et al., 2002). Later on, a null mutant in a gene closely related to *DAG1* and named *AtDof2.5/DAG2*, has been characterized by Gualberti et al. (2002). They showed that *DAG2* substantially reduced the capacity of seeds to germinate in the absence of light, while *DAG1* enhanced the dark germination (Gualberti et al., 2002). These two *Dof* TFs have therefore opposite effects during seed germination in response to light and are acting as repressor and activator respectively (Papi et al., 2000, 2002; Gualberti et al., 2002). Interestingly both these transcription factors are expressed specifically in the vascular system but neither in the seed or in the embryo as one could expect (Gualberti et al., 2002; Papi et al., 2002) suggesting that they indirectly control long-distance light related signaling pathways involved in embryo and seed development.

Two other *Dof* TFs, *AtDof1.5/COG1* and *AtDof3.6/OBP3*, have been shown to impact the seedling morphogenesis and especially the hypocotyl elongation which is also a light signaling related process. Park et al. (2003) showed that the activation tagged mutant *cog1-D*, in which *AtDof1.5*/*COG1* gene is overexpressed, displays a longer hypocotyl prominently under red and far-red lights. They suggested that *COG1* is a negative regulator in the phyA- and phyB-signaling pathways (Park et al., 2003). On the contrary, *OBP3* gene, which accumulates in the vascular tissues during the light period, is suggested to act as a positive regulator of the phyB-signaling pathway (Ward et al., 2005). In addition, Ward et al. (2005) showed that *OBP3* negatively regulate the cryptochrome-mediated seedling photomorphogenesis.

### AtDof TFs potentially control the phloem sugar transport

In *Arabidopsis*, a plant species that mainly transports sucrose for long-distance allocation of carbohydrates, the transition from sink-leaf to source-leaf is paralleled by the expression of *AtSUC2* that encodes a companion-cell (CC) specific H^+^-sucrose symporter (Truernit and Sauer, 1995; Stadler and Sauer, 1996). In 2008, an elegant 5′-deletion experiment combined with an *in silico* analysis of the At*SUC2* promoter, allowed identifying several *cis*-regulatory sequences in the minimal promoter sequence required to drive *AtSUC2* vascular expression, including three Dof-binding motifs and one motif recognized by HD-ZIP transcription factor (Schneidereit et al., 2008). Only the simultaneous deletion of at least one Dof-binding motif and the HD-ZIP binding site resulted in the loss of *AtSUC2* promoter activity, suggesting that a *Dof* TF in coordination with an HD-ZIP regulates the expression of the *SUC2* gene. So far no *Dof* TFs involved in *AtSUC2* transcriptional regulation have been identified in *Arabidopsis*. However, it has been clearly established that the ZmDof1 and ZmDof2 from maize control the expression of genes involved in carbon metabolism (Yanagisawa, 2001). Their two orthologs in *Arabidopsis AtDof1.4* and *AtDof3.5* could thus be proposed to regulate *AtSUC2* expression, which would give new insights in the fine-tuning of the sink-source transition.

## Toward an Integrated Transcriptional Network for the Control of Vascular Development and/or Functioning

Structurally, like for other zinc fingers, the Dof domain is known to be a bi-functional domain that mediates not only DNA-binding but also protein–protein interactions (Yanagisawa, 2002). When looking at *cis*-elements present in Dof TFs promoters, one can observe that each of the promoter displays at least one *cis*-element related to other transcription factor (bHLH, MADS boxes, ARF, Homeobox, MYB, bZIP, WRKY) (for more details, see the AGRIS interface, http://arabidopsis.med.ohio-state.edu/AtcisDB/). Altogether these data suggest that interactions between Dof TFs and TFs belonging to other families are theoretically possible. Experimentally, OBP1 was shown to directly interact with the promoter of a bZIP transcription factor but also with the promoter of a member of the same family *AtDof2.3* (Zhang et al., 1995; Kang and Singh, 2001; Skirycz et al., 2008). Moreover Kang et al. (2003) showed that two bHLH members (bHLH038 and bHLH039) are targets of the vascular expressed OBP3 transcription factor. Interestingly, bHLH039 was also found to be highly expressed in phloem tissue during hypocotyl secondary growth in *Arabidopsis* (Zhao et al., 2005). In the regulation of leaf axial patterning, a direct binding of AtDof5.1 on the promoter of the HD-ZIP, *REVOLUTA* have also been demonstrated (Kim et al., 2010). Besides these interactions at the transcriptional level, protein–protein interactions between the Dof TFs, CDF1, CDF2, and CDF3 and the F-box FKF1 have also been observed in the frame of the photoperiodic flowering response (Imaizumi et al., 2005). These few examples illustrate the fact that Dof TFs and TFs of other families (bHLH, bZIP, HD-ZIP, ARF, F-box) have the potential to interact together at the transcriptional as well as at the protein level and therefore be part of transcriptional networks involved in different developmental processes such as vascular development. Such transcriptional network, involving a bHLH complex, has been recently shown to control the establishment of the embryonic vascular tissue (De Rybel et al., 2013). This discovery opens a new level of complexity in the molecular mechanisms underlying the vascular development. Similar complexes involving Dof TFs could also be speculated. Indeed besides TARGET OF MONOPTEROS (TMO5/bHLH135), a Dof TF (AtDof5.3/TMO6) has been also identified as a potential indirect target of MP transcription factor (Schlereth et al., 2010) and has been described as one of the transcription factors participating in *Arabidopsis* vein formation (Gardiner et al., 2010). TMO6 could therefore be a good candidate to explore the involvement of Dof TFs in the regulation of the early steps of procambium formation. Altogether, TMO6 as well as the other Dof TFs mentioned in this review could constitute the starting point to study the potential implication of the Dof TFs in the transcriptional networks underlying vascular development.

## Conclusion and Future Prospects

In the context of the vascular system development and functioning, which integrates a large variety of internal or external stimuli, regulators are needed to fine-tune the responses to changes in the environment. In this review, we present evidences that the Dof TFs family is likely to participate in such fine-tuning of the responses to the different stimuli in the vascular system. By modulating a variety of transcription factors, by protein-DNA and/or protein–protein interactions, the Dof TFs could therefore act at the cross-talk of various developmental pathways directly or indirectly linked to the vascular development and/or functioning. One of the next challenges, besides the functional characterization of the still undescribed Dofs TFs, will therefore be identifying the actors involved in an integrated regulatory network. Combination of the high-throughput data collection at the resolution of the vascular cell types and developmental stages as well as the use of the next generation sequencing technology will certainly allow identifying genes network regulating the fundamental features of the vascular development.

## Conflict of Interest Statement

The authors declare that the research was conducted in the absence of any commercial or financial relationships that could be construed as a potential conflict of interest.
